# P-120. Treatment of Acute Uncomplicated Diverticulitis among Hospitalized Patients: Clinical Practices and Outcomes

**DOI:** 10.1093/ofid/ofae631.326

**Published:** 2025-01-29

**Authors:** Hussein Tehaili, Zainab Shams, Meenal Malviya, Susan M Szpunar, Leonard B Johnson

**Affiliations:** Ascension St. John Hospital, Dearborn Heights, Michigan; Ascension Macomb Hospital, Warren, Michigan; Ascension St. John Hospital, Dearborn Heights, Michigan; Ascension St. John Hospital, Dearborn Heights, Michigan; Ascension St. John Hospital, Dearborn Heights, Michigan

## Abstract

**Background:**

There are limited data comparing the outcomes of different antibiotic regimens for uncomplicated diverticulitis among hospitalized patients. We evaluated physician prescribing and outcomes based on discharge antibiotic regimens among hospitalized patients with acute uncomplicated diverticulitis.

**Methods:**

We performed a retrospective chart review on adult patients at a tertiary care hospital between July 2018 and June 2023 diagnosed with acute uncomplicated diverticulitis. Uncomplicated diverticulitis was defined as patients without perforation, peritonitis, abscess, or strictures. We collected data on demographics, comorbid medical conditions, Charlson score, imaging findings and antibiotic treatment regimens as inpatient and outpatient. Ninety-day outcome measures included readmissions for relapse of diverticulitis, intervention for complications (operative or by interventional radiology) and episodes of *Clostridioides difficile* infection (CDI). Data were analyzed using Student’s t-test, the chi squared test, and the Mann-Whitney U test.

**Results:**

Among 108 evaluable patients, 91.7% (99) received inpatient beta-lactam (BL) based therapies. Upon discharge, 44.4% (48) received fluoroquinolones (FQ) and 55.5 % (60) got BL based therapies. The groups were similar in demographics and clinical conditions; however, the median Charlson score was significantly higher in the BL group than the FQ group (1.0 (range 0-11) vs. 0.0 (range 0-7)), respectively. There were no differences in length of treatment as inpatient (3.3 days ±1.6 BL vs 3 days ± 1.3 FQ, p = 0.24) or outpatient (7.5 ± 4.6 BL vs. 7.0 ± 2.1 FQ, p = 0.51). Patients with a recurrence had a higher prevalence of rheumatologic disease than those without a recurrence, 28.6% vs. 9.6%, respectively, p=0.04), There was no significant difference in recurrence or readmission by outpatient regimen (57.1% BL vs 55.3% FQ, p = 0.9). There were two patients in the BL group who subsequently needed intervention and drainage on readmission and two episodes of CDI, one in each group.

**Conclusion:**

In our study, there was no overall difference in 90-day outcomes for patients with acute uncomplicated diverticulitis who received FQ or BL therapy on discharge. Further studies should be performed to confirm this observation.

**Disclosures:**

**All Authors**: No reported disclosures

